# Insulin Signaling and Dietary Restriction Differentially Influence the Decline of Learning and Memory with Age

**DOI:** 10.1371/journal.pbio.1000372

**Published:** 2010-05-18

**Authors:** Amanda L. Kauffman, Jasmine M. Ashraf, M. Ryan Corces-Zimmerman, Jessica N. Landis, Coleen T. Murphy

**Affiliations:** 1 Lewis-Sigler Institute for Integrative Genomics, Princeton University, Princeton, New Jersey, United States of America; 2 Department of Molecular Biology, Princeton University, Princeton, New Jersey, United States of America; Cold Spring Harbor Laboratory, United States of America

## Abstract

Of all the age-related declines, memory loss is one of the most devastating. While conditions that increase longevity have been identified, the effects of these longevity-promoting factors on learning and memory are unknown. Here we show that the *C. elegans* Insulin/IGF-1 receptor mutant *daf-2* improves memory performance early in adulthood and maintains learning ability better with age but, surprisingly, demonstrates no extension in long-term memory with age. By contrast, *eat-2* mutants, a model of Dietary Restriction (DR), exhibit impaired long-term memory in young adulthood but maintain this level of memory longer with age. We find that *crh-1*, the *C. elegans* homolog of the CREB transcription factor, is required for long-term associative memory, but not for learning or short-term memory. The expression of *crh-1* declines with age and differs in the longevity mutants, and CREB expression and activity correlate with memory performance. Our results suggest that specific longevity treatments have acute and long-term effects on cognitive functions that decline with age through their regulation of rate-limiting genes required for learning and memory.

## Abstract 2

Novel *C. elegans* associative learning and memory assays reveal that insulin/IGF-1 signaling and dietary restriction pathways differentially maintain age-related memory decline by influencing expression levels of the transcription factor CREB.

## Author Summary

In humans, aging is often associated with a decline in cognitive function. Progress toward an understanding of the molecular mechanisms underlying the initiation and progression of age-related neuronal decline could be hastened by the development of experimental systems that quickly test early and true symptoms (rather than the correlative downstream effects) of neuronal decline and disease. In contrast to muscle degradation, the nervous system of *C. elegans* is structurally remarkably well-preserved, leaving open the question of how to define age-related changes in neuronal function. To address this problem, we have established a novel system to study associative learning, short-term associative memory, and long-term associative memory in *C. elegans*. Through chemotaxis assays, we measured worms' ability to learn a positive association of a neutral chemoattractant with food. We found that long-term, but not short-term, associative memory is dependent on *crh-1*, the *C. elegans* homolog of the transcription factor CREB. Furthermore, we find that worm learning and long-term associative memory decreases with age and is influenced differently by insulin/IGF-1 and Dietary Restriction longevity pathways. These effects can be largely attributed to changes in expression of *crh-1*, which correlate with memory performance.

## Introduction

A guiding proposition of longevity research is that treatments that extend survival will also be generally beneficial to the health of the organism. However, many specifics of this concept remain to be tested. In humans, aging is often accompanied by declines in cognitive function. An understanding of the molecular mechanisms underlying the initiation and progression of age-related neuronal decline requires an experimental system to quickly test early symptoms, rather than the correlative downstream effects, of neuronal decline and disease. Although *C. elegans*' neural system is relatively simple compared with higher organisms, it has been an important model system for the study of neuronal development, synapse formation and function, and neuron-mediated behaviors. *C. elegans* has also been invaluable in the study of aging, revealing several longevity-modifying pathways that have proven to be conserved in higher organisms [1]–[6]. *C. elegans* is particularly useful as a model of post-mitotic cellular aging; because the cells do not turn over, maintenance of neuronal function must be due to cell and protein maintenance with age, as is the case for the majority of human brain cells. With its short lifespan and simple stereotyped nervous system, a *C. elegans* model characterizing the age-related neuronal decline of neurodegenerative disease may allow the identification of novel therapeutic targets for the earliest-onset cognitive disorders in humans.

Electron microscopy studies reveal that while *C. elegans* muscle tissue degrades with age, neuronal cells maintain their structural integrity [7]. However, this may not indicate a retention of function with age: humans display short-term memory loss that appears to be independent of neuronal degeneration [8]. Functional studies show that *Drosophila* also experience declines in olfaction and olfactory learning with age [9]. *C. elegans* displays age-related declines in chemotaxis [10] and isothermal tracking, a type of associative memory recalling the temperature at which an animal was raised. However, these declines significantly overlap with age-related declines in motility and may be related to degradation of muscle function [10]. Age-related decline in habituation (desensitization to mechanical stimulus) occurs late in adulthood as well, also overlapping with declines in muscle function [11]. Thus, it remains to be determined whether *C. elegans* experiences early age-related declines in higher-order neuronal function despite the structurally intact appearance of neurons.

Two of the primary regulators of longevity, Insulin/IGF-1 Signaling (IIS) and Dietary Restriction (DR), have been well-studied in *C. elegans*. The DAF-2 insulin receptor (WBGene00000898) and its downstream target, the transcription factor DAF-16/FOXO (WBGene00000912), regulate survival, stress resistance, and the maintenance of youthful movement in *C. elegans* [1],[12],[13]; its homologs in other organisms, including humans, also regulate aging, suggesting significant conservation of this pathway's functions [4],[5],[14]. The *C. elegans* mutant *eat-2* (WBGene00001133) is a model of Dietary Restriction and lives up to 50% longer than wild type [2]; DR increases survival in every organism tested [15]. Low insulin signaling in *daf-2* mutants maintains isothermal tracking and chemotaxis abilities with age better than wild type [10],[16]; conversely, high insulin levels decrease locomotion and spatial memory in mice [17], suggesting that insulin signaling's effects on cognition may also be conserved. DR has also been suggested to attenuate age-related cognitive decline [18], but the molecular bases for such effects are not yet known.

Here we have designed positive olfactory associative assays to measure *C. elegans* learning and memory. We have found that *C. elegans* long-term associative memory (LTAM) requires the same molecular components, such as transcription, translation, and CREB activity, as long-term memory in other organisms. Our aging results suggest that long-term olfactory memory is the first function to be lost with age and that olfactory learning, chemotaxis, and motility decline later and sequentially, prior to any obvious structural defects. We then tested these behaviors in the insulin-signaling and DR longevity mutants, both in young and aged worms, and found that these mutations have surprisingly different effects on age-related declines in learning and memory. We find that CREB levels and activity correlate well with long-term memory, suggesting an underlying molecular mechanism determining memory performance. Our results suggest that the regulation of the degeneration or maintenance of these behaviors may be conserved in higher organisms and may also be manipulable through specific longevity treatments.

## Results

### *C. elegans* Remember a Food-Odorant Association

**Figure 1:**
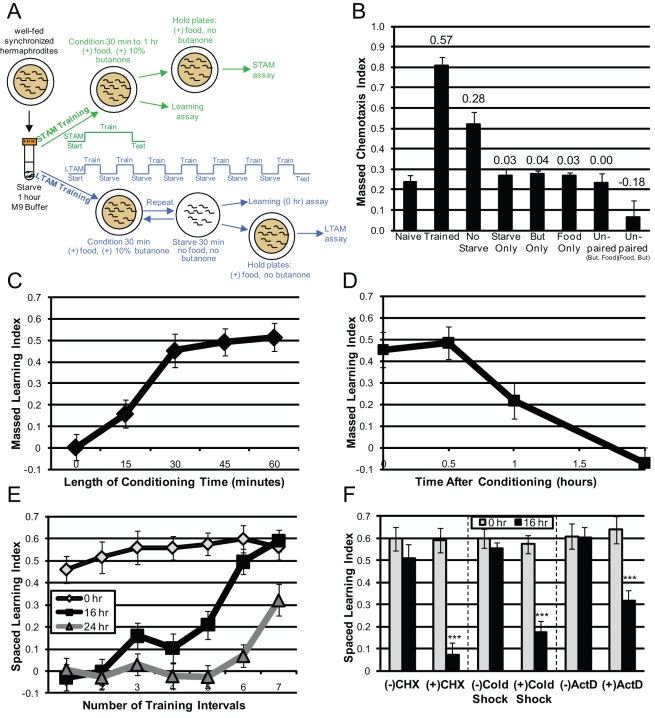
*C. elegans* learn and remember a positive association between food and the weak chemoattractant butanone. (A) Positive associative olfactory learning and memory assays. Well-fed worms are starved, then fed in the presence of 10% butanone; testing immediately after a single (massed) training measures learning, and short-term associative memory (STAM) is measured after an interval without exposure to butanone. Long-term associative memory (LTAM) is measured after several intervals of (spaced) training. (B) *C. elegans* positive associative learning. Worms were tested for chemotaxis toward 10% butanone before (Naïve) or after conditioning massed training (Trained). Control conditioning paradigms include conditioning training without the 1 h pre-starve, starving for 1 h alone, conditioning training with food alone, 10% butanone alone, or unpaired (butanone then food or food then butanone). Chemotaxis Index (CI)  =  (# at Butanone − # at Control)/(Total # − # at Origin). Learning Index (shown above bars) is calculated by subtracting the naïve CI from the post-conditioning training CI. (C) Wild-type 1× massed learning is saturated after 30 min of conditioning training with food and butanone. (D) Short-term memory of butanone association is lost by 2 h. (E) 16 h and 24 h LTAM increases with number of spaced training trials and requires butanone during spaced training (Figure S1E). (F) Disruption of protein synthesis (cycloheximide or cold shock) or transcription (actinomycin D) decreases LTAM but does not affect spaced learning performance. (B–F): *n* = 6 trials; ± SEM; *** *p*<0.001.

To examine cognitive decline in *C. elegans*, we developed simple Pavlovian appetitive associative learning and memory assays using the AWC neuron-sensed odorant butanone (Figure 1), and tested these behaviors with age in wild-type animals and in longevity mutants. Briefly, after a short starvation, worms are fed in the presence of butanone at a concentration that normally elicits a low chemotactic response (similar to Toroyama et al. [19]; Figure 1B), and then are tested for their attraction to butanone (Figure 1A). We found that after a single (“massed”) training, wild-type animals' chemotaxis to butanone increased ∼0.6 chemotaxis index units, which is its “Learning Index” (LI). This massed associative learning was saturated by 30 min of training (Figure 1C) and was dependent on the simultaneous presence of food and butanone during training (Figure 1B).

Memory can be separated into distinct classes based on duration and molecular requirements; in *Aplysia*, *Drosophila*, and mice, short-term memory lasts minutes to hours [20],[21], while long-term memory lasts hours to days and requires new protein synthesis and gene transcription [22]. To assess the duration of the learned association, we held worms on a plate with food but no butanone after a single training session. We found that the memory of the food-butanone association was retained less than two hours (Figure 1D), which is similar to the duration of *C. elegans* salt-starvation association [23]. Starvation after massed training only slightly extended this short-term associative memory (STAM) (Figure S1A).

In flies, mice, and *Aplysia*, training paradigms in which conditioning stimuli are presented to animals several times with rest periods between presentations (“spaced training”) yield longer-lasting memory than does massed training [22]. We found that spaced training also greatly enhanced the duration of *C. elegans*' memory of the food-butanone association: while the number of training blocks did not affect initial (“spaced”) learning (0 h, Figure 1E), recall increased with the number of training blocks. After seven training blocks, the learning index 16 h post-training was the same as that immediately after conditioning (Figure 1E). (Although the 16 h time point is arbitrary, it is similar to the time frame used in mammalian long-term memory studies [24].)

In our spaced-training paradigm, worms are starved in the “rest” period between conditioning training sessions and put onto food after training (the post-conditioning period). Therefore, any decline in LI after training is not due to adaptation since butanone (the conditioned stimulus) is not present between training and testing for memory. In terms of classical conditioning, holding worms on food after spaced training may be considered to be re-exposure to the unconditioned stimulus; however, in our assays it is critical to return animals to food, the neutral state, after conditioning, since significant transcriptional changes in response to starvation can occur as soon as 1 h after the removal of food [25]. Moreover, we find that holding naïve worms on plates without food for 16 h greatly increases their attraction to butanone (Figure S1B). Thus, starving the worms during the post-conditioning period would not allow a fair test of how well the association between butanone and food is retained.

Previous studies in *Drosophila* have demonstrated that varying the duration of the rest period during spaced training (either mechanically or through genetic manipulation) can affect recall performance [26],[27],[28]. We found that doubling or halving the length of time between training intervals appears to have no effect on long-term memory (Figure S1C).

Long-term memory in other organisms requires gene transcription and protein synthesis [22]. We found that cycloheximide treatment and cold shock, which interrupt protein synthesis, and actinomycin D treatment, which interrupts transcription, all abrogated 16 h memory but had no effect on the 0 h LI (Figure 1F), indicating that both protein translation and gene transcription are required for long-term memory but not for spaced learning. Thus, our spaced-training memory paradigm greatly enhances the duration of recall compared with the massed-training paradigm, and meets the transcriptional and translational requirements of classical long-term associative memory (LTAM).

### *C. elegans* Long-Term Associative Memory Requires the Transcription Factor CREB

**Figure 2:**
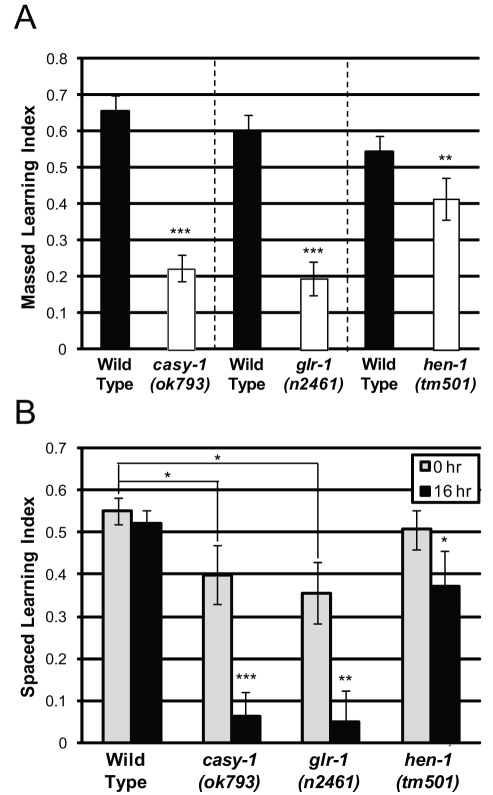
Known learning mutants perform poorly in learning and long-term memory assays. (A) *casy-1(ok793)*, *glr-1(n2461)*, and *hen-1(tm501)* mutants have significant learning defects after 1× massed training. (B) *casy-1(ok739)* and *glr-1(n2461)* mutants are defective for initial 0 h spaced learning and subsequent 16 h memory; *hen-1(tm501)* mutants are slightly defective for 16 h memory. (A–B): *n*≥6 trials (Figure S1D); ± SEM; * *p*<0.05, ** *p*<0.01, *** *p*<0.001.

Several genes that are required for olfactory learning have been identified, including *casy-1* (WBGene00000403), a calsyntenin [29]; *glr-1* (WBGene00001612), an AMPA-type glutamate receptor [30],[31]; and *hen-1* (WBGene00001841), a secretory protein required for cue integration and olfactory learning [32]. We found that these mutants performed normally in benzaldehyde chemotaxis assays (Figure S2A); however, these animals, especially *casy-1* and *glr-1*, exhibited defects in massed learning (Figure 2A) and long-term (16 h) memory (Figure 2B).

**Figure 3:**
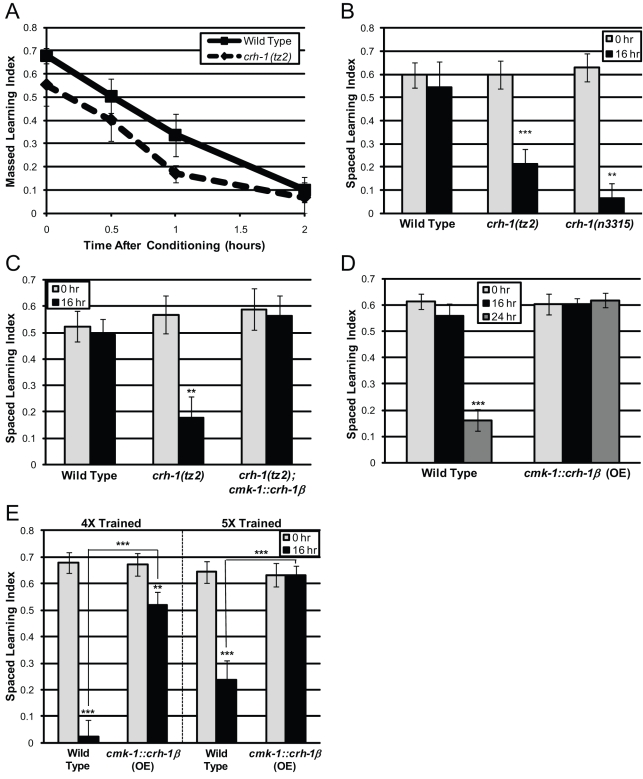
CREB (*crh-1*) is required for LTAM but not for learning or short-term memory. (A) Massed learning (0 h) and STAM (slope) is similar between wild-type and *crh-1(tz2)* mutant worms. (B) CREB (*crh-1(tz2)* and *crh-1(n3315)*) mutants learn after spaced training but are defective for long-term memory. (C) LTAM defect of *crh-1(tz2)* mutants is rescued by neuronal expression of CREB (*cmk-1::crh-1β*). (D) Overexpression of CREB in neurons (*cmk-1::crh-1β*) enhances LTAM performance after 7× training. (E) Animals overexpressing CREB form 16 h LTAM faster than wild type. (A–B, D–E): *n* = 6 trials; (C): *n* = 4 trials; ± SEM; ** *p*<0.01, *** *p*<0.001.

By contrast, we found that CREB, a bZIP transcription factor required for long-term memory in *Aplysia*, *Drosophila*, and mammals [22], is required specifically for LTAM: deletion allele mutants of CREB (*crh-1*, WBGene00000793) had normal benzaldehyde chemotaxis (Figure S2B), massed learning (Figure 3A), short-term memory (Figure 3A), and spaced learning (Figure 3B), but were defective for long-term memory (Figure 3B). In fact, *crh-1* recall is lost by 2 h post-spaced training (), while wild type shows no decrease at this point, highlighting the requirement for CREB activity in long-term memory. Expression of CREB under a neuronal-specific promoter (*crh-1(tz2);cmk-1::crh-1β* [33]) completely rescued the long-term memory defect of the *crh-1(tz2*) deletion mutant (Figure 3C). Moreover, neuronal overexpression of CREB in a wild-type background both increases long-term memory duration (Figure 3D) and reduces the number of training sessions to achieve 16 h memory (Figure 3E). Together, our results suggest that learning is molecularly distinct from but required for subsequent memory, and that CREB is specifically required for long-term associative memory.

### Learning and Memory Decline with Age

**Figure 4:**
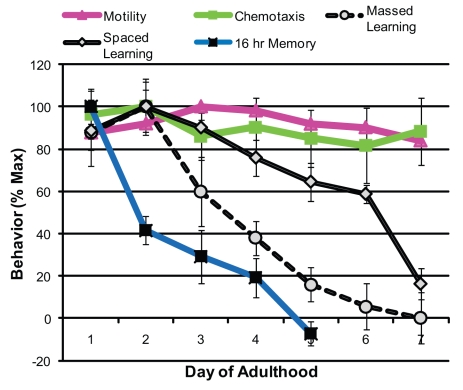
Learning and memory behaviors display the earliest age-related decline. During the first week of adulthood, motility and chemotaxis ability are maintained, while 1× massed and 7× spaced learning and long-term associative memory abilities are lost by Day 7 and Day 5 of adulthood, respectively. *n* = 6 trials (except Mobility, *n* = 1 trial); ± SEM.

The observation that *C. elegans* neurons do not display obvious age-dependent structural degeneration [7] leads to the question of whether worms experience functional neuronal decline. Thus, we tested the effect of aging on various neuronally-controlled behaviors. While motility and chemotaxis were unaffected through the first week of adulthood (as shown previously [10],[34]), we found that massed learning, spaced learning, and long-term memory abilities declined quickly (Figure 4). Strikingly, 16 h long-term memory was impaired significantly by Day 2–3 and was completely lost by Day 5. Thus, not only do learning and long-term memory require different gene activities (Figures 2, 3), but these behaviors also decline at different rates, suggesting that the molecularly distinct mechanisms of learning and memory are also differently susceptible to aging.

### Reduced Insulin Signaling Improves Memory in Young Adults

**Figure 5:**
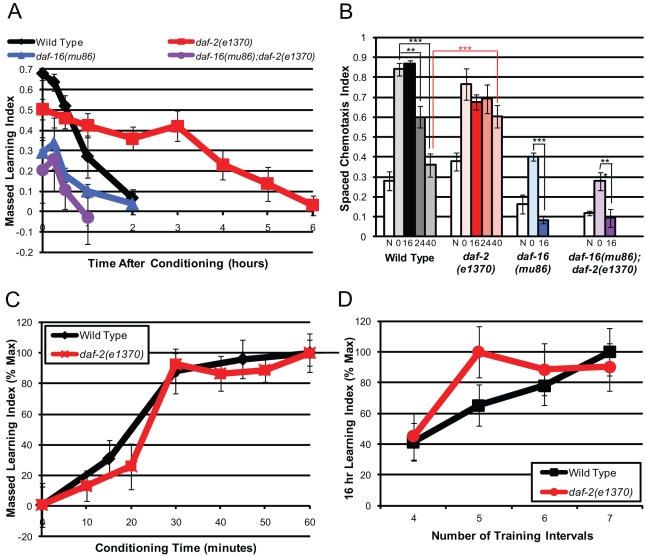
Insulin signaling mutants increase Day 1 adult memory. (A) After massed training, *daf-2(e1370)* mutants retain short-term memory more than three times as long as wild type, in a *daf-16*-dependent manner. (B) After spaced training, *daf-2(e1370)* mutants maintain long-term associative memory significantly longer than wild type, also dependent on *daf-16*. N  =  naïve, numbers under bars represent hours after 7× spaced training. (C) *daf-2(e1370)* mutants learn at the same rate as wild-type after massed training. (D) *daf-2(e1370)* mutants form 16 h memory faster with spaced training than wild-type. (A–D): *n* = 6 trials; ± SEM; ** *p*<0.01, *** *p*<0.001.

*daf-2* insulin/IGF-1 receptor mutants are long-lived and morphologically youthful [1] and thus might be predicted to maintain cognitive abilities with age. In our positive appetitive assay, *daf-2* mutants displayed no defects in chemotaxis to butanone or to another AWC-sensed odorant, benzaldehyde (Figure S2B), consistent with *daf-2* performance in chemotaxis adaptation assays [10], and no learning defects (Figure S3A). Strikingly, *daf-2(e1370)*, *daf-2(e1368)*, and *daf-2(RNAi)* animals (Figure 5A,B; Figure S3B–E) displayed greatly increased duration of memory on the first day of adulthood: *daf-2*'s short-term memory lasted more than 3 times as long as wild type's (Figure 5A, Figure S3B,C), and *daf-2*'s long-term memory at 40 h is still more than 60% of its initial learning levels (Figure 5B, Figure S3D,E). *daf-2*'s short- and long-term memory extensions both require the activity of the downstream transcription factor *daf-16/*FOXO (Figure 5A,B).

Are *daf-2* worms simply less plastic, acquiring and losing information more slowly than wild-type worms do? To answer this question, we measured the rate of learning in both the massed and spaced-training paradigms. In the massed training paradigm, *daf-2* worms learned at a rate similar to wild type, with maximum learning achieved after 30 min of conditioning (Figure 5C), suggesting that *daf-2*'s basic massed learning ability is similar to wild type's. However, *daf-2* worms established LTAM faster than wild type, reaching maximum 16 h memory with only five training blocks (Figure 5D), similar to the performance of CREB overexpression animals (Figure 3E). These results suggest that reduced insulin signaling does not change plasticity but can both establish the long-term memory of an association more quickly and prolong the duration of this association.

### Dietary Restriction Impairs Young Adult Memory

**Figure 6:**
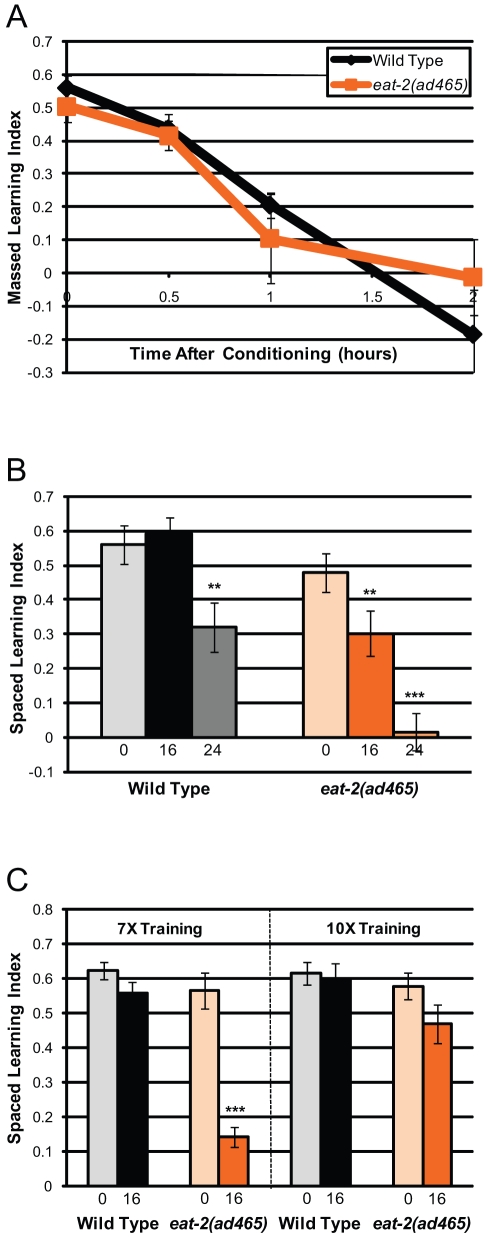
Dietary restriction impairs Day 1 long-term memory. (A) *eat-2(ad465)* mutants have normal massed learning and short-term memory. (B) *eat-2(ad465)* mutations impair long-term memory after spaced training. (C) Increasing spaced training blocks from seven to ten improves the defective 16 h memory of *eat-2(ad465)* animals. Numbers under bars represent hours after 7× spaced training. (A–C): *n* = 6 trials, ± SEM; ** *p*<0.01, *** *p*<0.001.

*daf-2*'s cognitive phenotypes could be specific to IIS or could be general for all longevity pathways. To differentiate these possibilities, we examined the acetylcholine receptor mutant *eat-2*, a model of the well-established DR longevity mechanism. *eat-2* encodes a nicotinic acetylcholine receptor (nAChR) that functions postsynaptically in pharyngeal muscle to regulate the rate of pharyngeal pumping [35],[36]. *eat-2* mutants ingest food (*E. coli*) poorly and extend life span through a *daf-16*-independent DR pathway [2],[37]. We found that Day 1 adult *eat-2* mutants displayed normal benzaldehyde chemotaxis (Figure S2B) and normal massed learning (Figure 6A, Figure S4A), suggesting that its decreased food ingestion does not affect its ability to form food-olfactory associations or to chemotax toward odorants. *eat-2*'s short-term memory duration was the same as wild type's (Figure 6A, Figure S4A), in contrast to *daf-2*'s significant STAM extension (Figure 5A, Figure S4B,C). However, in two point mutation allele mutants, *eat-2* animals' long-term memory was significantly impaired, with a complete abrogation of memory by 24 h (Figure 6B, Figure S4B). *eat-2*'s neutral effect on STAM and negative effect on LTAM were unexpected, based on our observations that starvation extends STAM (Figure S1A) and that *daf-2* mutations extend both STAM and LTAM (Figure 5A,B, –E). Increasing the number of training blocks from seven to ten improves *eat-2*'s LTAM to a level similar to wild type's after 7× spaced training (Figure 6C), suggesting that *eat-2* mutants can form long-term memories but require more training to do so.

**Figure 7:**
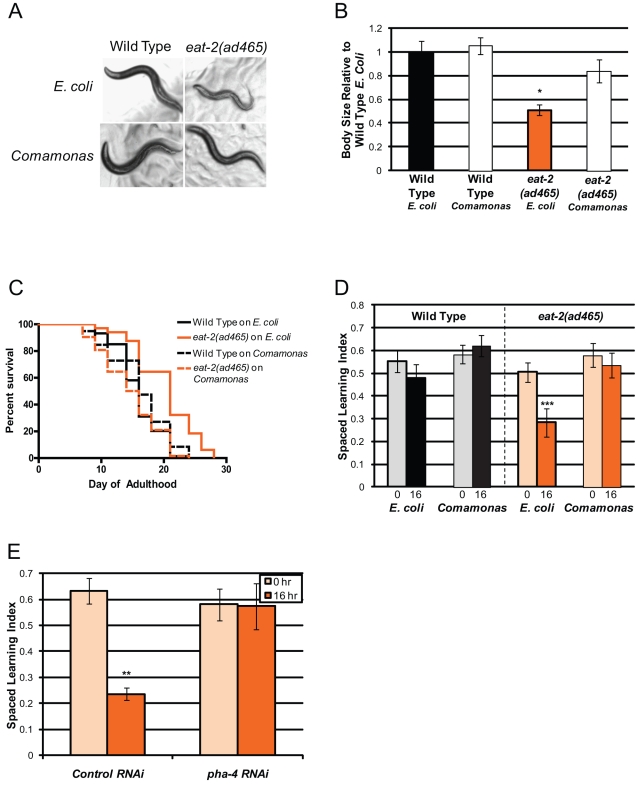
Feeding *eat-2* worms with *Comamonas sp.* rescues Dietary Restriction phenotypes. (A) When fed standard food of *E. coli*, *eat-2(ad465)* mutants exhibit a small body size compared to wild type. *eat-2(ad465)* mutant body size is rescued by feeding animals small bacteria (*Comamonas*). (B) Quantitation of body size represented in (A). (C) The extended lifespan of *eat-2(ad465)* mutants is suppressed by feeding with *Comamonas*. (D) Feeding with *Comamonas* rescues *eat-2(ad465)* animals' 16 h memory defect but does not significantly affect wild type's memory. (E) Treatment of *eat-2(ad465)* worms with *pha-4* RNAi abolishes LTAM defect. (B): *n* = 3 Day 1 adult worms; photos are at same magnification; (C): *n*>70, WT/*E. coli* versus *eat-2*/*E. coli*: *p*<0.001; versus *eat-2*/*Comamonas*: *p* = 0.43; versus WT/*Comamonas*: *p* = 0.11; (D–E): Numbers under bars represent hours after 7× spaced training; (D): *n* = 6 trials; (E) *n* = 3 trials; ± SEM; * *p*<0.05, ** *p*<0.01, *** *p*<0.001.

To rule out the acetylcholine receptor itself as the source of *eat*-2's memory impairment, we fed *eat-2* mutants smaller, “easier to eat” bacteria, *Comamonas sp.* [38] (Leon Avery, personal communication). *Comamonas* had no effect on the growth or longevity of wild-type worms but suppressed *eat-2*'s small size and long life span (Figure 7A–C, Figure S4C); thus, these worms still had the mutant acetylcholine receptor but were not dietarily restricted. Strikingly, *Comamonas* also suppressed *eat-2*'s long-term memory defect (Figure 7D). All of *eat-2*'s phenotypes were also suppressed by treatment with RNAi of *pha-4*, the FoxA transcription factor that mediates *eat-2*'s effects on longevity (Figure 7E) [2],[37]. Together, these results suggest that the memory impairment we observe in *eat-2* mutants is indeed due to DR rather than to acetylcholine receptor dysfunction. Thus, while Dietary Restriction and reduced insulin signaling both increase longevity, the two pathways influence cognitive ability of young adults in an opposite manner.

### Reduced Insulin Signaling and Dietary Restriction Differentially Affect Maintenance of Learning and Memory with Age

**Figure 8:**
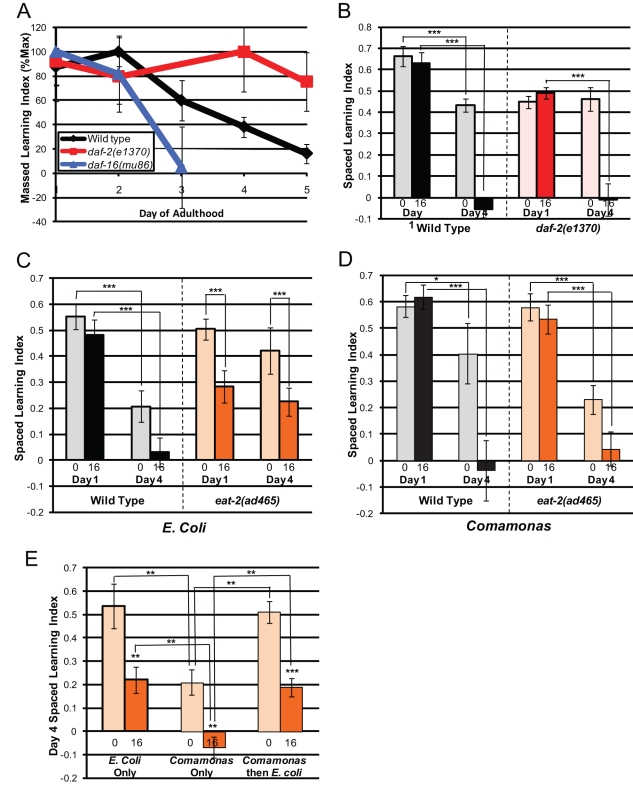
Reduced insulin signaling and Dietary Restriction affect maintenance of learning and memory with age differently. (A) *daf-2(e1370)* worms retain the ability to learn with massed training longer with age. (B) *daf-2(e1370)* animals learn better after spaced training than wild type on Day 4 of adulthood but do not display improved long-term memory with age. (C) *eat-2(ad465)* maintain spaced learning and memory with age, which is suppressed by feeding with *Comamonas* (D). (E) Post-developmental induction of Dietary Restriction improves maintenance of spaced learning and memory on Day 4 of adulthood. *eat-2(ad465)* worms were cultivated on *Comamonas* until Day 1 of adulthood, then switched to growth on *E. coli*. (B–D): Numbers under bars represent hours after 7× spaced training; (A–D): *n* = 6 trials; (E) *n* = 4 trials; ± SEM; * *p*<0.05, *** *p*<0.001.

To test the roles of IIS and DR in the maintenance of cognitive ability with age, we measured *daf-2* and *eat-2* mutants' learning and memory abilities later in adulthood. We found that *daf-2* mutants retain the ability to learn longer than do wild-type or *daf-16* worms, with no significant loss in massed learning ability at Day 5, when wild-type massed learning ability has completely ceased (Figure 8A, Figure S5A). Surprisingly, however, *daf-2* mutants lose long-term memory with age at the same rate as wild type: on Day 4, *daf-2* mutants had better spaced learning than wild-type worms but exhibited no significant 16 h memory (Figure 8B). Thus, despite extending longevity and learning ability with age, reduced insulin signaling does not appear to maintain memory performance with age.

Like *daf-2*, *eat-2*'s learning ability is maintained with age: on Day 4, *eat-2* mutants learned better than Day 4 wild-type worms after spaced training (Figure 8C). However, in contrast to *daf-2* mutants, *eat-2* mutants maintain both short- and long-term memory with age, as Day 4 *eat-2* animals exhibited no significant decline from their performance on Day 1 (Figure 8C, Figure S5B,C). This maintenance of long-term memory can be attributed to DR, as *Comamonas* feeding suppressed both the aged learning and memory phenotypes of *eat-2* mutants (Figure 8D).

To determine whether DR strictly in adulthood can rescue age-related memory phenotypes, we raised *eat-2* animals on *Comamonas* until early adulthood, then switched them to *E. coli* to induce DR. When switched post-developmentally, the animals were still large and exhibited normal (wild-type-like) Day 1 memory (Figure S5D–F) but retained Day 4 memory better than wild-type (Figure 8E, Figure S5G), suggesting that memory loss was alleviated by DR.

Thus, while DR and reduced IIS both increase longevity, the two pathways have very different effects on cognitive behaviors, both early in adulthood and with age.

### Memory Performance Correlates with CREB Transcriptional Levels and Activity

**Figure 9:**
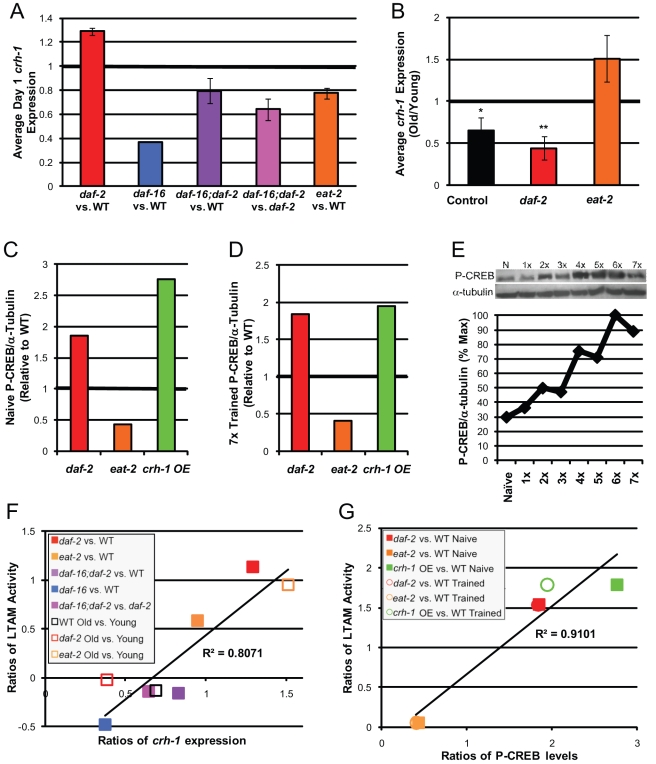
CREB/*crh-1* expression and P-CREB activity correlate with memory performance. (A) CREB (*crh-1*) expression is increased in *daf-2* and decreased in *daf-16*, *daf-16;daf-2*, and *eat-2(ad465)* mutant worms relative to wild type on Day 1 of adulthood. (B) *crh-1* expression significantly declines in wild-type and *daf-*2 worms with age but does not decline in *eat-2* worms. (C–D) P-CREB levels are higher in *daf-2* mutant and *crh-1*-overexpressing worms and lower in *eat-2* mutant worms both before (C) and after (D) 7× LTAM training, relative to wild type. (E) P-CREB levels increase with LTAM training. (F) *crh-1* expression levels correlate with LTAM activity in longevity mutants and with age (R^2^ = 0.81). (G) P-CREB levels correlate with LTAM activity in longevity mutants and *crh-1-*overexpressing worms on Day 1 of adulthood (R^2^ = 0.91). (A–B, F): *n*≥4 (except *daf-16* expression, *n* = 1); ± SEM; * *p*<0.05, ** *p*<0.01, *** *p*<0.001. (C–D, G): Western blots shown in Figure S6C,E.

To identify the underlying molecular mechanisms that might distinguish IIS and DR effects on long-term memory maintenance with age, we examined the transcriptional levels of key learning and memory genes. While the expression of the learning genes *glr-1* and *casy-1* did not change significantly with age (Figure S6A), we found that CREB/*crh-1* expression levels correlate with memory performance: *crh-1* levels are higher in young *daf-2* than in wild-type (Figure 9A) or *daf-16;daf-2* animals, lower in young *eat-2* than in wild-type (Figure 9A), fall with age in both wild-type and *daf-2* worms (Figure 9B), and are maintained with age in *eat-2* mutants (Figure 9B, Figure S6B).

To determine whether changes in CREB transcriptional levels reflect changes in activity, we used an anti-phosphorylated CREB antibody to assay levels of activated CREB. First, we found that naïve *crh-1* overexpression worms have a higher level of phosphorylated CREB (P-CREB) than did wild-type animals (Figure 9C, Figure S6C). Secondly, both wild-type and CREB overexpression worms showed increased P-CREB levels post-training (Figure 9D, Figure S6C); these levels increased with training sessions (Figure 9E, Figure S6D), parallel to LTAM activity (Figure 1E). Increases in CREB activity with training sessions also parallels the LTAM performances of *daf-2* and *eat-2* mutant worms (Figures 5, 6): while *daf-2* P-CREB levels increased fairly linearly through six training trials (Figure S6F), P-CREB levels increased only with additional training sessions in *eat-2* animals (Figure S6G).

To determine how well *crh-1* expression levels and LTAM activity correlate, we plotted the ratios of LTAM activities against the ratios of *crh-1* levels for eight pairs of samples (Figure 9F); the R^2^ value of 0.8 indicates that *crh-1* ratios are a reasonable predictor of relative LTAM activity. An even stronger correlation was found when we plotted the ratios of LTAM activities against ratios of P-CREB levels (R^2^ = 0.9; Figure 9G). Thus, CREB activity appears to be the major molecular mechanism determining LTAM performance, and *crh-1* levels may be an excellent predictor of long-term memory performance.

## Discussion

While it is known that many neuronal structures remain intact with age [7],[8], previously it was not clear how higher-order neuronal functions are affected by aging. While basic motor skills and chemotaxis abilities continue through later stages of adulthood, we find that higher-level cognitive abilities are lost much earlier in adulthood. Our assays are able to distinguish between the processes of massed learning, spaced learning, short-term memory, and long-term memory, and our results suggest that not only do these processes have distinct molecular requirements, but they also decline differentially with age. We find that long-term memory, which inherently requires learning and chemotaxis abilities, declines particularly early in adulthood, prior to the decline of learning, chemotaxis, and motility. LTAM likely involves complex synaptic machinery [20] and thus may be particularly susceptible to age-related damage. Associative olfactory learning and memory appear to be more sensitive to age-related decline than other behaviors, such as isothermal tracking [10] or habituation [11]. Interestingly, anosmia is recognized as one of the earliest symptoms of neurodegeneration, including Alzheimer's and Parkinson's disease [39], and declines in taste are linked to olfactory defects [40]. Therefore, food-smell associations may be extremely effective in evaluating changes in learning and memory with age and neurodegeneration in humans as well as in worms.

We have also demonstrated for the first time, to our knowledge, the requirement of CREB activity in *C. elegans* memory. The differential effects of the IIS and DR pathways on learning and memory decline with age appear to be attributable to their differential regulation of CREB/*crh-1* expression levels and activity, rather than to changes in learning-associated genes such as *glr-1*, *casy-1*, and *hen-1*. In general, we find that CREB expression level changes can largely account for the decline in wild-type memory with age and its maintenance in longevity mutants (Figure 9), suggesting that CREB levels may be a good indicator of long-term memory function. CREB levels and activity also decline with age in the mammalian brain [41],[42], and over-expression of CREB in the hippocampus increases the performance of aged animals in several long-term memory tasks [43]. Thus, the molecular mechanisms underlying *C. elegans* long-term memory, particularly CREB's importance, are likely conserved in higher organisms. Our results imply that specific types of longevity treatments could have either positive or negative effects on learning and memory, and therefore, it will be crucial to examine the effects of specific longevity treatments on maintenance of human cognitive behaviors with age.

## Materials and Methods

### Worm Cultivation

Animals were cultivated at 20°C on HGM plates on OP50 *E. coli* or *Comamonas sp.* (DA1877) using standard methods [44] and developmentally synchronized by hypochlorite treatment. Worms were moved to HGM + 50 mM FUdR at the L4 stage when tested for learning or memory after Day 1 of adulthood.

### Strains

Wild type: (N2 Bristol); mutant strains: RB888 (*casy-1(ok739)*), KP4 (*glr-1 (n2461)*), JC2154 (*hen-1(tm501)*), DA465 (*eat-2(ad465)*), DA1116 (*eat-2(ad1116*)), CF1041 (*daf-2(e1370)*), CF1038 (*daf-16(mu86)*); CF1043 (*daf-16(mu86);daf-2(e1370)*); MT9973 (*crh-1(n3315)*); YT17 (*crh-1(tz2)*); and YT50 (*crh-1(tz2);cmk-1::crh-1β*). The *tz2* mutation lacks 979 nucleotides/38 residues at the C-terminus of CREB's bZIP region, and no functional protein is expressed [33]. Alkema and Horvitz report that *n3315* is a loss of function deletion allele that eliminates the expression of all four *crh-1* isoforms (Wormbase). The “*crh-1* OE” strain (CQ71) was made by crossing N2 with YT50 animals and selecting worms carrying the *cmk-1::crh-1β* transgene.

### Chemotaxis Assay

Chemotaxis assays were performed according to previously described methods [45]. >200 developmentally-synchronized worms were placed at origin, and the number at butanone (1 µL 1:10 butanone:ethanol + 1 µL NaN_3_), ethanol control (+ 1 µL NaN_3_), and origin were counted after 1 h. Chemotaxis Index (CI)  =  [(n_attractant_) − (n_control_)] / [(Total − n_origin_)].

### Mobility Assay

Mobility was measured on each day of adulthood by calculating the percentage of worms that remained at the origin of a chemotaxis assay plate after 1 h.

### Learning Assay (1× Massed Training)

Synchronized Day 1 adult hermaphrodites were starved in M9 buffer for 1 h, transferred to a 60 mm NGM plate with 500 µL freshly-seeded OP50 or DA1877 and 2 µL of 10% butanone on lid, trained for 1 h, then tested for chemotaxis to butanone. LI  =  CI_Trained_ − CI_Naive_.

### STAM Assay

After 1× massed training, worms were transferred to 60 mm NGM plates freshly seeded with 500 µL OP50 or DA1877 (“holding plate”) for specified intervals.

### LTAM Assay

After 1 h of starvation, worms received seven training blocks (30 min on training plates with food and butanone, followed by two M9 washes and 30 min on plates without food). Worms were then tested immediately for spaced learning (“0 h”) or transferred to holding plates for 16 or 24 h. SEM and student's *t* test was used to assign *p* values in all assays.

### Protein Synthesis and Transcription Inhibition during LTAM Training

Protein synthesis inhibition: animals were cold shocked at −20°C [30] for 15 min, then were returned to the conditioning temperature (20°C) for 15 min, or treated with 800 µg/mL cycloheximide [46], during the starvation period of each training block. Transcription inhibition: animals were treated with 100 µg/mL Actinomycin D during the starvation period of each training block [47].

### Survival Analysis

Wild-type or *eat-2(ad465)* worms were cultivated and life span assays were carried out at 20°C on NGM + 50 µM FUdR with OP50 (*E. coli*) or DA1877 (*Comamonas sp.*). The first day of adulthood was defined as *t* = 0. *n*>70 for each strain. Standard Kaplan-Meier survival analysis was used to assess significance ([48], GraphPad, Prism 5.01).

### RNAi

RNAi clones were PCR-verified. RNAi-sensitive *eri-1(mg366);lin-15B(n744)* (*daf-2* RNAi) or *eat-2(ad465)* (*pha-4* RNAi-treated) animals were synchronized and cultivated on vector control or RNAi bacteria on NGM plates with 0.1 M IPTG (final concentration) at 20°C until Day 1 of adulthood.

### Gene Expression Analysis

Data for gene expression analyses with age in wild-type, *daf-2*, and *daf-16;daf-2* conditions was provided by Murphy et al. [49]. *eat-2(ad465)* and *daf-16(mu86)* mutants were collected and analyzed as previously described [49],[50]. Data were filtered for quality, and replicates were collapsed to an average value (PUMAdb; http://puma.princeton.edu). RT-PCR was carried out to verify expression results (Figure 9A, Figure S6B). cDNA was made from total worm RNA (checked for 230/260/280 quality before processing) using TaqMan Reverse Transcription Reagents (Applied Biosystems). Serial dilutions of 0.5 µg/mL cDNA were used in 20 µL PCR reactions. For *crh-1* RT-PCR experiments, the primers used (forward: ATGTCAGCGAAAGGTAACGG, reverse: CGTTTTGTTGTGGTCCTCCT) amplify a 442 bp fragment located at 30–471 bp in the 1,197 bp mRNA sequence (NCBI reference sequence NM_001027690.1), a region that lies upstream of the deletion described for *crh-1(tz2)*.

### Western Blot Analysis

Worms were washed in M9, collected, and frozen in liquid nitrogen. Lysates were prepared by freeze/thawing worm pellets in lysate buffer (50 mM HEPES, 1 mM EDTA, 150 mM NaCl, 1 mM NaFl, 10% glycerol, 1% Triton X-100, proteinase inhibitor), and sonication to break cells. Protein concentrations were quantified using Coomassie Plus (Pierce). Anti-Phospho-CREB (Ser133) rabbit mAb (Cell Signaling Technology 87G3, #9198) was used to probe for P-CREB; Anti-α-Tubulin mouse mAb (Sigma-Aldrich, #T9026) was used as a probe for the loading control, as we were unable to find a working antibody for total CREB in *C. elegans*. Antibodies were diluted 1:1,000 in 1× TBS-T, 5% BSA. Quantification of Western blot results was performed using “GeneTools” software from SynGene; P-CREB levels were compared to the α-tubulin loading control.

## Supporting Information

Figure S1 **Associative learning and memory controls.** (A) Post-conditioning 1× massed trained worms on holding plates without food increases short-term associative memory but still declines within hours. (B) Worms starved for 16 h have a significantly higher naïve chemotaxis to butanone than well-fed worms. (C) Halving (15 min) or doubling (60 min) the time of the starvation period during 7× training does not affect LTAM performance. (D) Replicates of wild type LTAM. Wild Type 1–4 spaced trained on OP50, Wild Type 5 grown and spaced trained on L4440 (Control vector) RNAi. 0 h and 16 h across all five sets of WT experiments was averaged for Figure 2B. (E) Spaced training with both food and butanone is required for the formation of 16 h memory. (A–C, E): *n*  =  6; (B) *n*  =  3 trials for WT 1 and 5, *n*  =  6 trials for WT 2–4; ± SEM; *** *p* < 0.001.
(3.00 MB TIF)

Figure S2 **Mutant benzaldehyde chemotaxis and** ***crh-1(tz2)*** **LTAM controls.** (A) Naïve learning mutants *casy-1(ok793)*, *glr-1(n2461)*, and *hen-1(tm501)* all chemotax normally to AWC-sensed odorant benzaldehyde (9.8%). (B) Naïve longevity mutants *daf-2(e1370)* and *eat-2(ad465)*, and CREB mutant *crh-1(tz2)* all chemotax normally to 9.8% benzaldehyde. (C) *crh-1(tz2)* 16 h memory is depleted by 4 h after LTAM spaced training. (A–C): *n*  =  6; ± SEM; * *p* < 0.05, ** *p* < 0.01.
(3.00 MB TIF)

Figure S3 **Insulin signaling learning and memory controls.** (A) *daf-2(e1370)* has higher naïve chemotaxis but still shows enhanced association between food and butanone after 7× spaced conditioning. (B–C) Like *daf-2(e1370)* worms (Figure 5A), *daf-2(e1368)* (B) and *daf-2(RNAi)* (C) animals also exhibit extended STAM on Day 1 of adulthood. (D–E) Like *daf-2(e1370)* worms (Figure 5B), *daf-2(e1368)* (D) and *daf-2(RNAi)* (E) animals also display extended LTAM on Day 1 of adulthood. N  =  naïve, numbers under bars represent hours after 7× spaced training. (A, D–E): *n*  =  6 trials; (B–C): *n*  =  4 trials; ± SEM; *** *p* < 0.001.
(3.00 MB TIF)

Figure S4 **Dietary Restriction learning and memory and lifespan controls.** (A) *eat-2(ad465)* worms have wild-type-like 1× massed learning and STAM. (B) Like *eat-2(ad465)* worms (Figure 6B), *eat-2(ad1116)* mutants also exhibit defective LTAM. Numbers under bars represent hours after 7× spaced training. (C) Feeding with *Comamonas* suppresses *eat-2(ad465)*'s lifespan extension phenotype. (A): *n*  =  6 trials; (B): *n*  =  4 trials; ± SEM; ** *p* < 0.01; (C): *n* > 70 animals; WT/*E. coli* versus *eat-2*/*E. coli*: *p* < 0.001; versus *eat-2*/*Comamonas*: *p*  =  0.25; versus WT/*Comamonas*: *p*  =  0.003.
(3.00 MB TIF)

Figure S5 **Controls for IIS and Dietary Restriction learning and memory with age.** (A) *daf-2(e1370)* mutants extend 1× massed learning with age, while *daf-16(mu86)*'s massed learning declines more quickly with age. (B) *daf-2(e1370)* STAM declines with age. (C) *eat-2(ad465)* STAM is maintained with age. (D) Day 1 adult *eat-2(ad465)* worms (Figure 8E) raised on *Comamonas* are significantly larger than those grown on *E. coli*. (E) Day 1 adult *eat-2(ad465)* worms (Figure 8E) raised on *Comamonas* have wild-type-like LTAM. (F) Day 1 adult *eat-2(ad465)* worms raised on *Comamonas* have wild-type-like LTAM compared to those grown on Control RNAi (antibiotic-selectable *E. coli*). (G) Post-developmental induction of Dietary Restriction improves maintenance of spaced learning and memory on Day 4 of adulthood. *eat-2(ad465)* worms were cultivated on *Comamonas* until Day 1 of adulthood, then switched to growth on Control RNAi (antibiotic-selectable *E. coli*). (A): *n*  =  1 trial; (B–C): *n*  =  6 trials; (D): *n* ≥ 15 worms; (E–G): *n*  =  4 trials. Numbers under bars represent hours after 7× spaced training; ± SEM; ** *p* < 0.01.
(3.00 MB TIF)

Figure S6 **Expression of learning and memory genes and P-CREB levels.** (A) Expression levels of learning genes *glr-1* and *casy-1* in old versus young wild-type worms. (B) Semi-quantitative RT-PCR verification of *crh-1* expression with age in wild-type, *daf-2(e1370)*, and *eat-2(ad465)* worms. (C) P-CREB levels increase after 7× training in wild-type and *crh-1*-overexpressing animals; P-CREB levels are higher in *crh-1*-overexpressing worms relative to wild type before and after 7× training (Figure 8C, D). (D) P-CREB levels increase in wild-type worms with 7× training. (E) P-CREB levels are higher in *daf-2(e1370)* and lower in *eat-2(ad465)* worms relative to wild-type before and after 7× training (Figure 8C, D). (F) P-CREB levels increase in *daf-2(e1370)* worms with 7× training. (G) P-CREB levels do not begin to increase in *eat-2(ad465)* worms until after six training blocks. (A–B): *n* ≥ 4; ± SEM; ** *p* < 0.001.
(3.00 MB TIF)

## Abbreviations

CI: Chemotaxis Index
CREB: cyclic-AMP Response Element Binding protein
DR: Dietary Restriction
IIS: Insulin/IGF-1 Signaling
LI: Learning Index
LTAM: Long-Term Associative Memory
P-CREB: phosphorylated CREB
STAM: Short-Term Associative Memory
