# Supplementary figures and images for: Insulin Signaling and Dietary Restriction Differentially Influence the Decline of Learning and Memory with Age
Source: PLoS Biol. 2010 May 18;8(5):e1000372. doi: 10.1371/journal.pbio.1000372 (PMC2872642; doi:10.1371/journal.pbio.1000372)

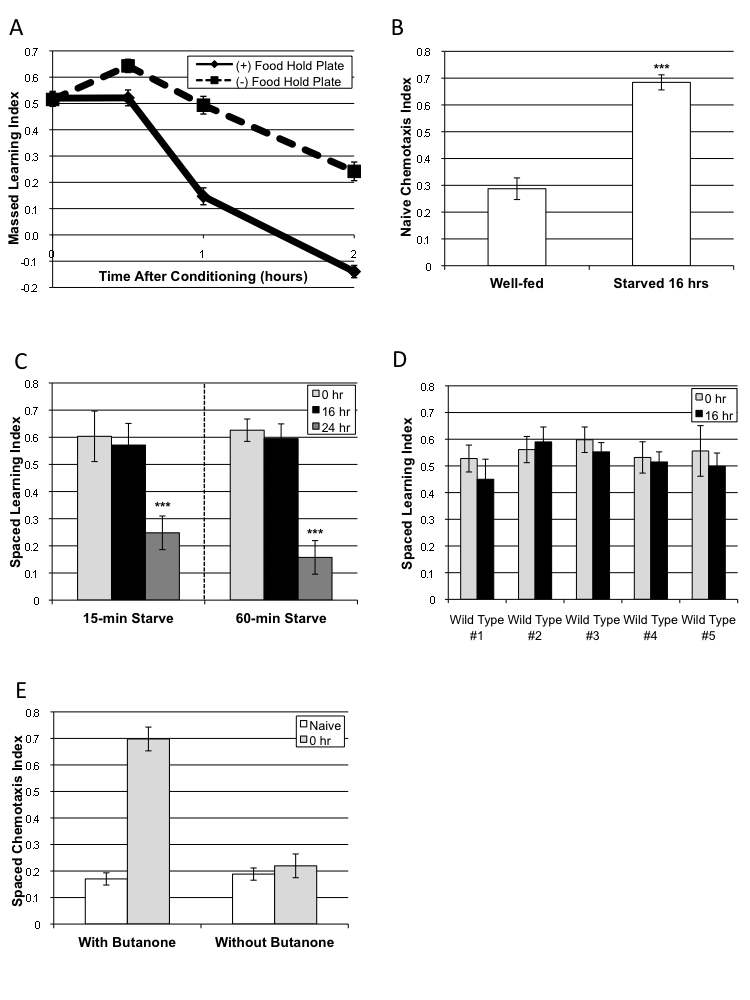

Supplement: Figure S1 — Associative learning and memory controls. (A) Post-conditioning 1× massed trained worms on holding plates without food increases short-term associative memory but still declines within hours. (B) Worms starved for 16 h have a significantly higher naïve chemotaxis to butanone than well-fed worms. (C) Halving (15 min) or doubling (60 min) the time of the starvation period during 7× training does not affect LTAM performance. (D) Replicates of wild type LTAM. Wild Type 1–4 spaced trained on OP50, Wild Type 5 grown and spaced trained on L4440 (Control vector) RNAi. 0 h and 16 h across all five sets of WT experiments was averaged for Figure 2B. (E) Spaced training with both food and butanone is required for the formation of 16 h memory. (A–C, E): n = 6; (B) n = 3 trials for WT 1 and 5, n = 6 trials for WT 2–4; ± SEM; *** p < 0.001. (3.00 MB TIF) [file pbio.1000372.s001.tif]

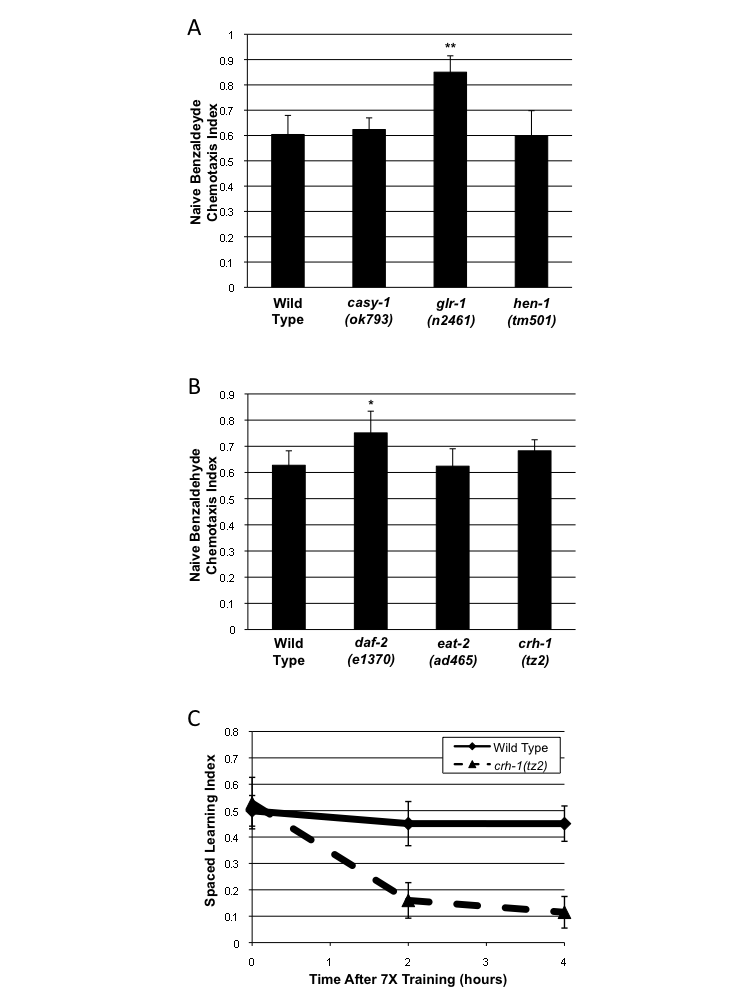

Supplement: Figure S2 — Mutant benzaldehyde chemotaxis and crh-1(tz2) LTAM controls. (A) Naïve learning mutants casy-1(ok793), glr-1(n2461), and hen-1(tm501) all chemotax normally to AWC-sensed odorant benzaldehyde (9.8%). (B) Naïve longevity mutants daf-2(e1370) and eat-2(ad465), and CREB mutant crh-1(tz2) all chemotax normally to 9.8% benzaldehyde. (C) crh-1(tz2) 16 h memory is depleted by 4 h after LTAM spaced training. (A–C): n = 6; ± SEM; * p < 0.05, ** p < 0.01. (3.00 MB TIF) [file pbio.1000372.s002.tif]

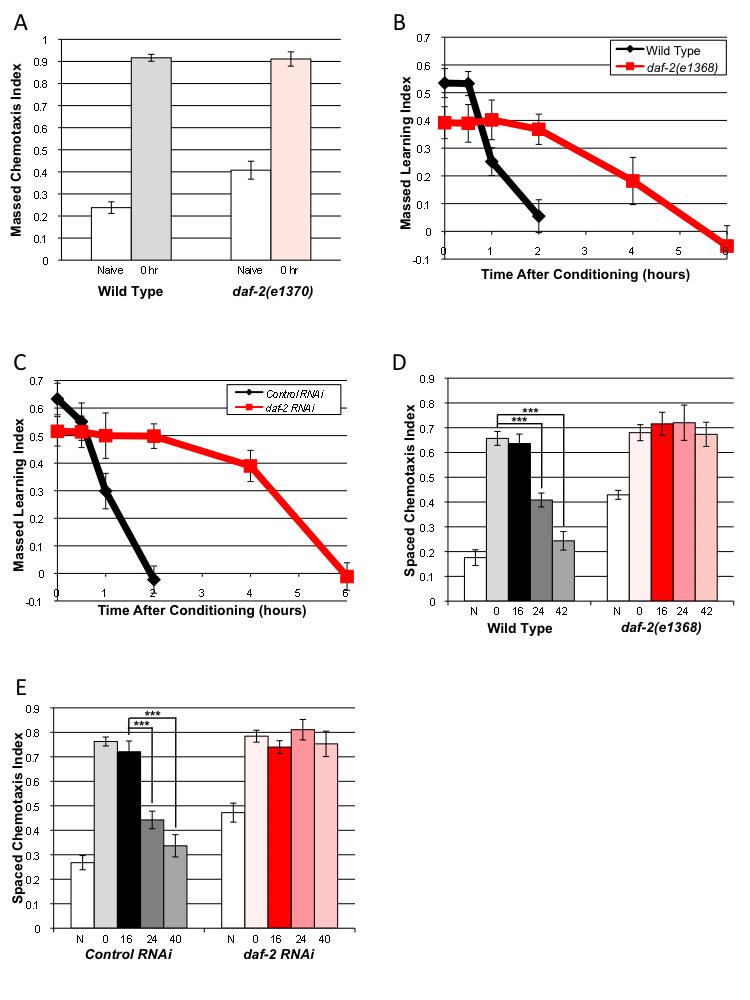

Supplement: Figure S3 — Insulin signaling learning and memory controls. (A) daf-2(e1370) has higher naïve chemotaxis but still shows enhanced association between food and butanone after 7× spaced conditioning. (B–C) Like daf-2(e1370) worms (Figure 5A), daf-2(e1368) (B) and daf-2(RNAi) (C) animals also exhibit extended STAM on Day 1 of adulthood. (D–E) Like daf-2(e1370) worms (Figure 5B), daf-2(e1368) (D) and daf-2(RNAi) (E) animals also display extended LTAM on Day 1 of adulthood. N = naïve, numbers under bars represent hours after 7× spaced training. (A, D–E): n = 6 trials; (B–C): n = 4 trials; ± SEM; *** p < 0.001. (3.00 MB TIF) [file pbio.1000372.s003.tif]

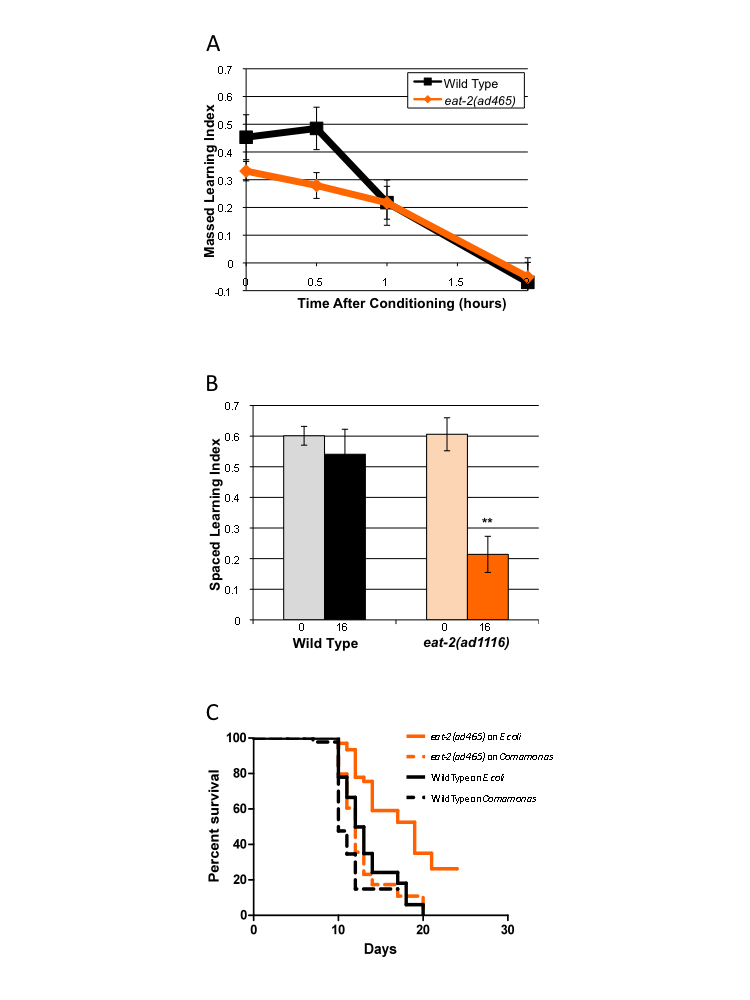

Supplement: Figure S4 — Dietary Restriction learning and memory and lifespan controls. (A) eat-2(ad465) worms have wild-type-like 1× massed learning and STAM. (B) Like eat-2(ad465) worms (Figure 6B), eat-2(ad1116) mutants also exhibit defective LTAM. Numbers under bars represent hours after 7× spaced training. (C) Feeding with Comamonas suppresses eat-2(ad465)'s lifespan extension phenotype. (A): n = 6 trials; (B): n = 4 trials; ± SEM; ** p < 0.01; (C): n > 70 animals; WT/E. coli versus eat-2/E. coli: p < 0.001; versus eat-2/Comamonas: p = 0.25; versus WT/Comamonas: p = 0.003. (3.00 MB TIF) [file pbio.1000372.s004.tif]

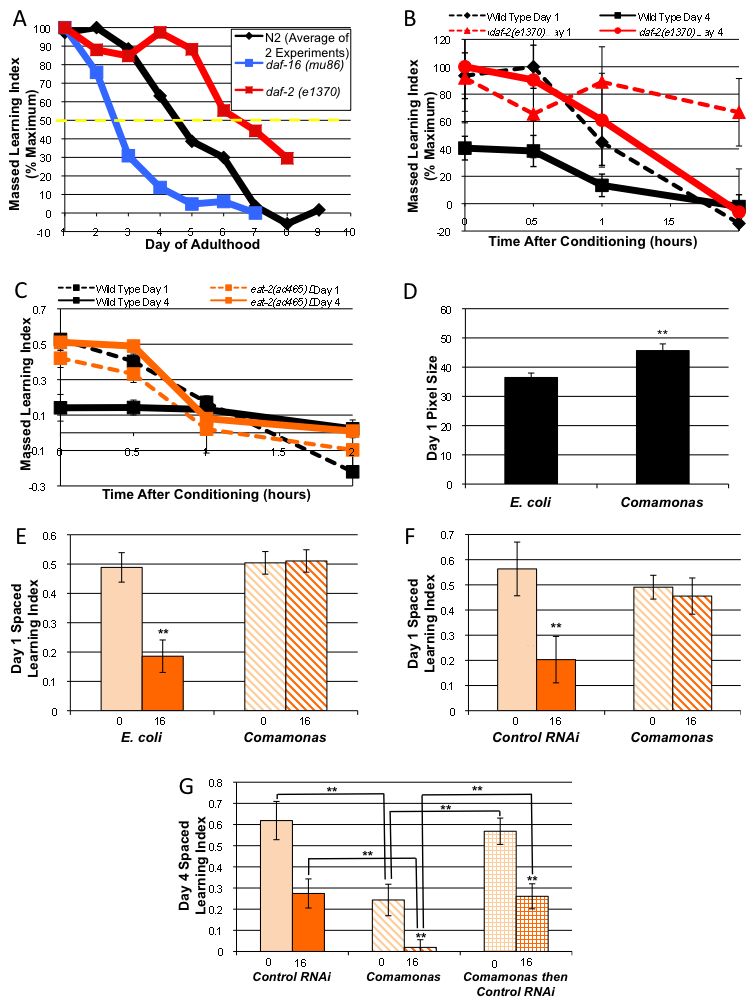

Supplement: Figure S5 — Controls for IIS and Dietary Restriction learning and memory with age. (A) daf-2(e1370) mutants extend 1× massed learning with age, while daf-16(mu86)'s massed learning declines more quickly with age. (B) daf-2(e1370) STAM declines with age. (C) eat-2(ad465) STAM is maintained with age. (D) Day 1 adult eat-2(ad465) worms (Figure 8E) raised on Comamonas are significantly larger than those grown on E. coli. (E) Day 1 adult eat-2(ad465) worms (Figure 8E) raised on Comamonas have wild-type-like LTAM. (F) Day 1 adult eat-2(ad465) worms raised on Comamonas have wild-type-like LTAM compared to those grown on Control RNAi (antibiotic-selectable E. coli). (G) Post-developmental induction of Dietary Restriction improves maintenance of spaced learning and memory on Day 4 of adulthood. eat-2(ad465) worms were cultivated on Comamonas until Day 1 of adulthood, then switched to growth on Control RNAi (antibiotic-selectable E. coli). (A): n = 1 trial; (B–C): n = 6 trials; (D): n ≥ 15 worms; (E–G): n = 4 trials. Numbers under bars represent hours after 7× spaced training; ± SEM; ** p < 0.01. (3.00 MB TIF) [file pbio.1000372.s005.tif]

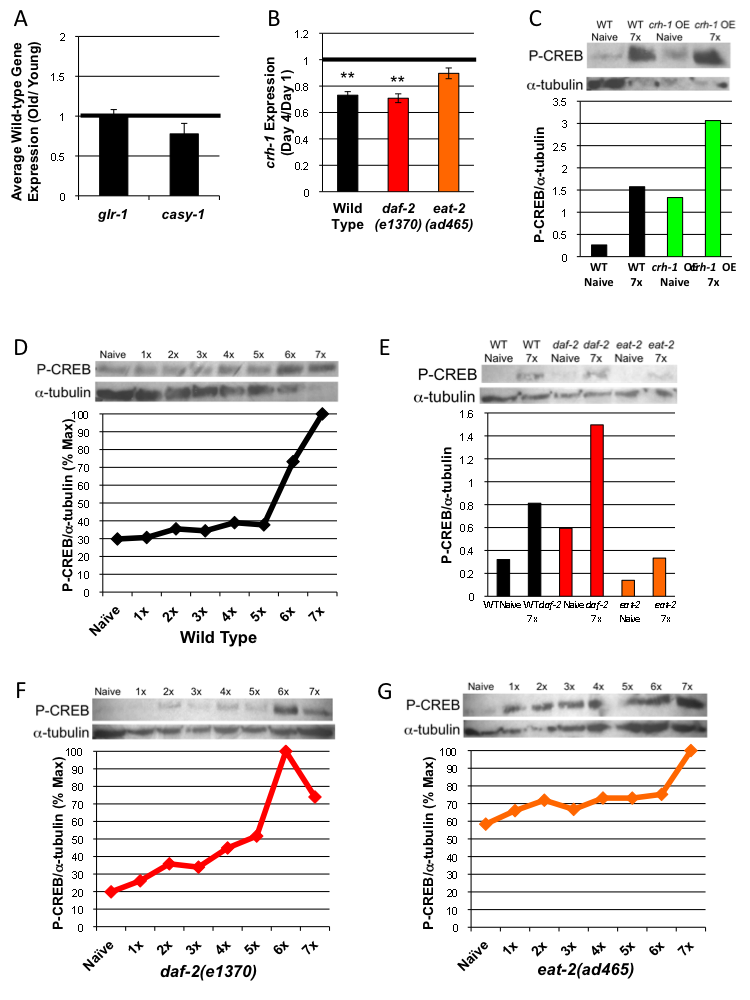

Supplement: Figure S6 — Expression of learning and memory genes and P-CREB levels. (A) Expression levels of learning genes glr-1 and casy-1 in old versus young wild-type worms. (B) Semi-quantitative RT-PCR verification of crh-1 expression with age in wild-type, daf-2(e1370), and eat-2(ad465) worms. (C) P-CREB levels increase after 7× training in wild-type and crh-1-overexpressing animals; P-CREB levels are higher in crh-1-overexpressing worms relative to wild type before and after 7× training (Figure 8C, D). (D) P-CREB levels increase in wild-type worms with 7× training. (E) P-CREB levels are higher in daf-2(e1370) and lower in eat-2(ad465) worms relative to wild-type before and after 7× training (Figure 8C, D). (F) P-CREB levels increase in daf-2(e1370) worms with 7× training. (G) P-CREB levels do not begin to increase in eat-2(ad465) worms until after six training blocks. (A–B): n ≥ 4; ± SEM; ** p < 0.001. (3.00 MB TIF) [file pbio.1000372.s006.tif]
